# Molecular tracking of individual host use in the Shiny Cowbird – a generalist brood parasite

**DOI:** 10.1002/ece3.2234

**Published:** 2016-06-12

**Authors:** Ma Alicia de la Colina, Mark E. Hauber, Bill M. Strausberger, Juan Carlos Reboreda, Bettina Mahler

**Affiliations:** ^1^ Departamento de Ecología, Genética y Evolución, and IEGEBA‐CONICET Facultad de Ciencias Exactas y Naturales Universidad de Buenos Aires Buenos Aires Argentina; ^2^ Department of Psychology Hunter College and the Graduate Center of the City University of New York 695 Park Avenue New York New York 10065; ^3^ School of Biological Sciences University of Auckland 3A Symonds Street PB 92019 Auckland New Zealand; ^4^ Pritzker Laboratory for Molecular Systematics and Evolution Field Museum of Natural History 1400 S. Lake Shore Drive Chicago Illinois 60605

**Keywords:** Host preference, laying patterns, microsatellites, *Molothrus bonariensis*, mtDNA, nest‐use strategies

## Abstract

Generalist parasites exploit multiple host species at the population level, but the individual parasite's strategy may be either itself a generalist or a specialist pattern of host species use. Here, we studied the relationship between host availability and host use in the individual parasitism patterns of the Shiny Cowbird *Molothrus bonariensis*, a generalist avian obligate brood parasite that parasitizes an extreme range of hosts. Using five microsatellite markers and an 1120‐bp fragment of the mtDNA control region, we reconstructed full‐sibling groups from 359 cowbird eggs and chicks found in nests of the two most frequent hosts in our study area, the Chalk‐browed Mockingbird *Mimus saturninus* and the House Wren *Troglodytes aedon*. We were able to infer the laying behavior of 17 different females a posteriori and found that they were mostly faithful to a particular laying area and host species along the entire reproductive season and did not avoid using previously parasitized nests (multiple parasitism) even when other nests were available for parasitism. Moreover, we found females using the same host nest more than once (repeated parasitism), which had not been previously reported for this species. We also found few females parasitizing more than one host species. The use of an alternative host was not related to the main hosts' nest availability. Overall, female shiny cowbirds use a spatially structured and host species specific approach for parasitism, but they do so nonexclusively, resulting in both detectable levels of multiple parasitism and generalism at the level of individual parasites.

## Introduction

Brood parasitic species exploit the parental care of other host species (Friedmann 1964, Payne 1977; Rothstein 1990; Davies 2000; Schulze‐Hagen et al. 2009). In order to reproduce successfully, among the most important decisions a brood parasitic female has to make include where, when and how many eggs to lay. Females of generalist brood parasitic species (i.e., that parasitize many hosts species) must make decisions to select both suitable host species (Teuschl et al. 1998; Hahn et al. 1999; Strausberger and Ashley 2003; Langmore and Kilner 2007; Strausberger and Rothstein 2009) and lay in nests of available individual hosts among suitable breeders (Soler et al. 1995; Hauber 2001; Polačiková et al. 2008; Fiorini et al. 2009; Soler and Pérez‐Contreras 2012). Additionally, parasitism must be synchronized with the host's laying cycle to maximize incubation schedules, timing of hatching, and subsequent chick survival (Davies and Brooke 1988; Hauber 2003; Ellison et al. 2006; Moskat et al. 2006; Fiorini et al. 2009).

**Figure 1:**
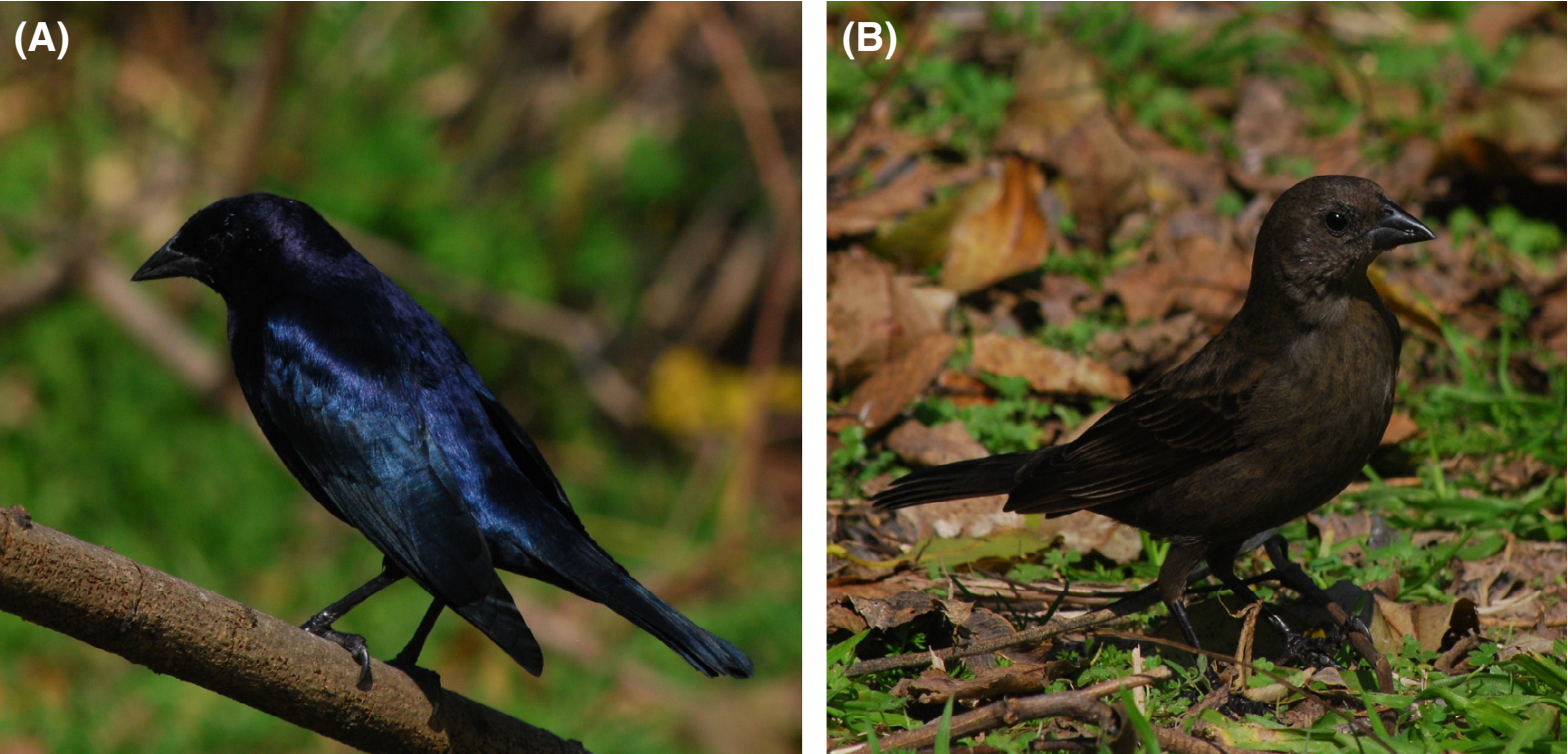
Male (A) and female (B) of Shiny Cowbird (Photos: F. Furiolo).

Individual host‐use strategies differ among generalist brood parasitic species. While in the common cuckoo *Cuculus canorus* each female parasitizes predominantly one host species (Marchetti et al. 1998; Skjelseth et al. 2004; Fossøy et al. 2011, 2016), evidence indicates there exist both host‐specialist and host‐generalist individuals within populations of both the brown‐headed cowbird *Molothrus ater* (Alderson et al. 1999; Woolfenden et al. 2003; Strausberger and Ashley 2005) and the bronzed cowbird *Molothrus aeneus* (Ellison et al. 2006). In these species, egg‐laying decisions are sometimes flexible and related to the availability and/or quality of hosts, being thus variable among and within breeding seasons (Woolfenden et al. 2003; Strausberger and Ashley 2005). Parasites should avoid the use of a host that has been already parasitized to prevent the competition with other parasitic chicks (Strausberger 1998; Hahn et al. 1999; Trine 2000; Hoover 2003; McLaren et al. 2003; Moskat et al. 2006; Goguen et al. 2011). However, if the host is able to raise more than one brood parasite, this intraspecific competition might be less costly than investing time and energy in searching for a different nest (Martínez et al. 1998). Multiple parasitism, whereby different females lay eggs in the same nest, is widespread among parasitic *Molothrus* cowbirds. Several studies have shown that although females defend territories (Hauber 2001), multiple parasitism is frequent in the brown‐headed cowbird with different females parasitizing the same nest (also called superparasitism) and/or the same parasitic female using one nest more than once (i.e., repeated parasitism; Alderson et al. 1999; Hahn et al. 1999; McLaren et al. 2003; Ellison et al. 2006; Hauber et al. 2012). Previous studies showed that multiple parasitism increases with density of parasitic females and reduced availability of host nests across different avian host–parasite systems (Strausberger 1998; Moskat et al. 2006; Rivers et al. 2012). In the shiny cowbird *Molothrus bonariensis* (Fig. 1), multiple parasitism is common (Lyon 1997; Ortega 1998; Gloag et al. 2012) and probably consequence of an absence of territoriality and defensive behaviors by parasitic females (Mermoz and Reboreda 2003; Scardamaglia and Reboreda 2014). However, no events of repeated parasitism have been reported yet for this species (Kattan 1997; Lyon 1997; Gloag et al. 2014).

The aim of this study was to analyze individual nest‐use strategies of the shiny cowbird and test whether parasitism strategies are related to hosts' nests availability. This obligate brood parasite is extremely generalist at population level, known to use the nests of more than 260 species (Friedmann and Kiff 1985; Lowther 2014). Indirect, genetic evidence based on population parameters suggests that females do however not lay their eggs randomly but preferentially parasitize certain host species and nest location types (López‐Ortiz et al. 2006; Mahler et al. 2007), which vary according to the particular host community that is being parasitized (De Mársico et al. 2010). Nonrandom host use by shiny cowbird females was supported by other findings, based on morphological differentiation between cowbird eggs laid in different colonies (Lyon 1997) or in different host nests (de la Colina et al. 2010; Tuero et al. 2012) and implied from behaviors like synchronization of laying and egg‐puncturing that vary according to the host (Fiorini et al. 2009). Here, for the first time, we studied the individual laying behavior of shiny cowbird females (Fig. 1B) and investigate host preference, nest selection, and temporal laying patterns using genetic tools. We analyze individual female's nest use and explore whether it is related to nest availability. We also analyze temporal laying ranges of individual females and examine whether individuals continue to lay throughout the breeding season or different females sequentially replace each other during the reproductive season. Knowing individual host‐use strategies in generalist brood parasites is crucial to understand coevolutionary interactions between parasites and hosts and the impact of parasitism on hosts' reproductive success and the viability of their populations. In the case of generalist females, parasitism can constitute a serious threat to less abundant or preferred hosts as parasite's population dynamics will mainly depend on the availability of abundant hosts and will not be affected by the contraction of less abundant ones. Therefore, these hosts can experience increases in parasitism frequencies although their populations are declining. On the contrary, if females are specialists, population dynamics of each “host‐specific race” will be associated in a density‐dependent way to this host and parasite's population number will be regulated by the host it uses (May and Robinson 1985; Ney‐Nifle et al. 2005). Discerning individual host‐use strategies will also improve the comprehension of coevolutionary interactions with host species and the selective pressures operating on them.

## Methods

### Study system

**Figure 2:**
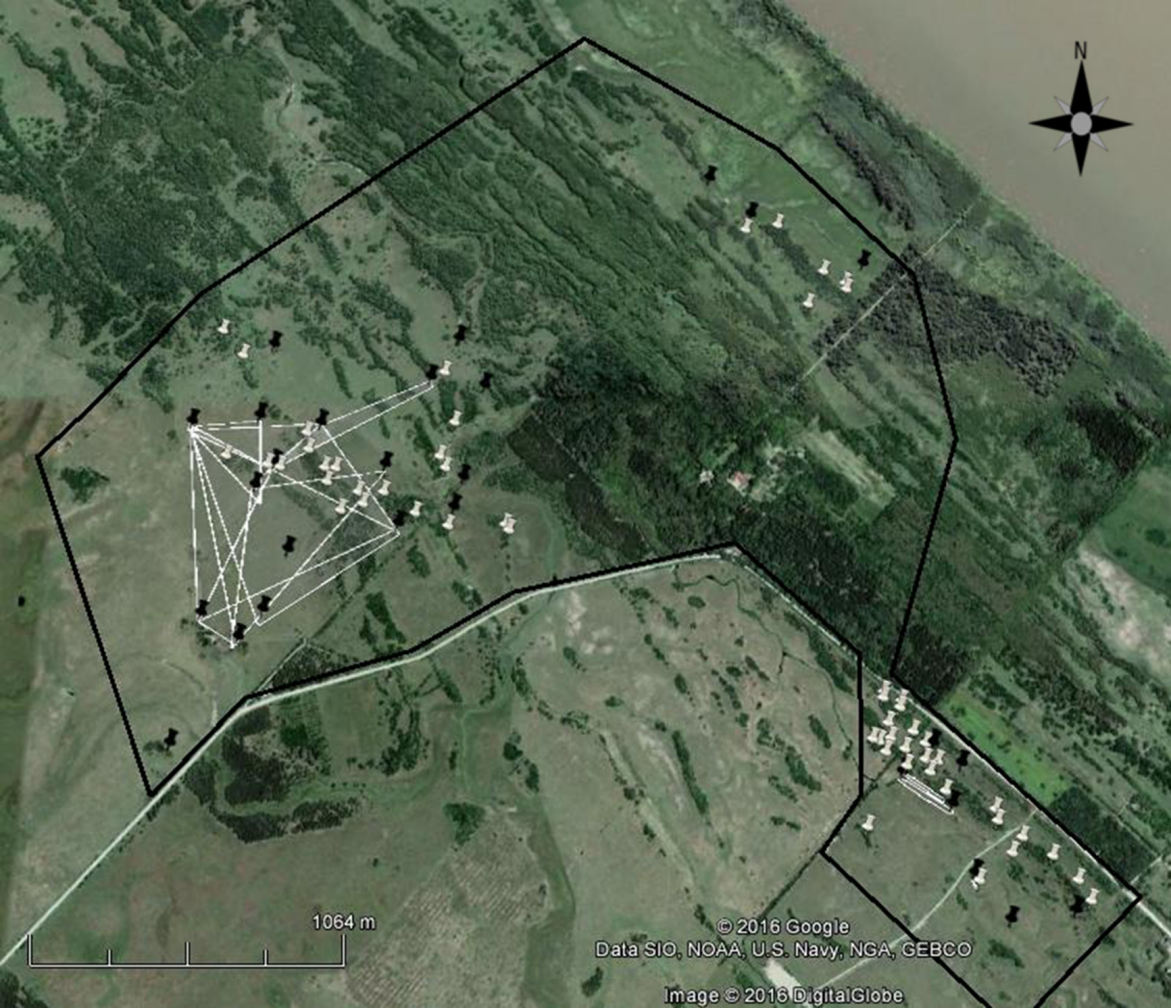
Study site with pinpointed geographical locations of all nests found in the year 2008; black pins correspond to nests of chalk‐browed mockingbirds (*N* = 77), white pins to house wren nests (*N* = 35). Polygons show maximal laying areas of the 10 females with full‐sibling offspring groups estimated for 2008 (see text). The black polygon shows the complete searching area.

We conducted the study at the private reserve “El Destino” (35°8′S, 57°23′W) near the town of Magdalena, Buenos Aires Province, Argentina. The study site is nearly flat, marshy grassland with interspersed woodland patches dominated by *Celtis ehrenbergiana* and *Scutia buxifolia*. We collected shiny cowbird eggs found in hosts' nests in an area of approximately 580 ha within the reserve. This area was not continuous but was subdivided into three plots separated by forest patches of exotic species (*Ligustrum* spp. and *Eucalyptus* spp.) where abundance of cowbird hosts is very low and one area used for other experiments by other members of our research group (Fig. 2). While there are various host species in this area (Mason 1986), two species are the main hosts: the chalk‐browed mockingbird *Mimus saturninus* (hereafter: mockingbird) and the house wren *Troglodytes aedon* (hereafter: wren) (De Mársico et al. 2010), with parasitism frequencies of 89% (Gloag et al. 2012) and 60% (Tuero et al. 2007), respectively. During their breeding seasons (October–January) of 2008–2009, 2009–2010, and 2010–2011, we systematically searched for the nests of these main hosts in the study area and collected cowbird samples (shiny cowbird eggs or blood samples of cowbird chicks). Other host species are also present and breeding in the study area, but they experience considerably lower parasitism frequencies (*Zonotrichia capensis* 25%, *Furnarius rufus* 20%, *Sicalis flaveola* 8%, *Agelaioides badius* 20%, Mason 1986); in the case of the two latter, they are also less abundant than the two main host species (Mason 1985), with only a couple of nests found within the patches used for sample collection. We nonetheless collected cowbird offspring samples from parasitized nests of these hosts that were found occasionally during nest searching and monitoring of mockingbird and wren breeding sites.

Mockingbirds lay three to five eggs, incubation starting with the laying of the penultimate egg and lasting ~14 days. Chicks leave the nest when they are 12–14 days old (Fiorini 2007). We searched for nests by focusing on adult mockingbird activity and then inspecting potential nesting sites within the territory of each breeding pair. Adults are extremely territorial and maintain their territories all year round (Fraga 1985) often using the same shrubs for nesting. It was possible to follow all breeding attempts of mockingbird pairs within our study area. We monitored nests daily during the periods of advanced construction and laying in order to detect parasitism events. Mockingbirds are partial accepters and only reject immaculate cowbird eggs within the first 24 h after parasitism (Fraga 1985; Mason 1986; Sackmann and Reboreda 2003, de la Colina et al. 2012). The immaculate eggs represent a low proportion of cowbird eggs in the study area (7%, Gloag et al. 2014). To reduce the probability of losing these cowbird eggs, mockingbird's nests were monitored mainly in the first hours of the morning.

Wrens have an average clutch size of five eggs, incubation starts with the laying of the penultimate egg and lasts ~15 days, nestling period is 16 days (Skutch 1953; Tuero et al. 2007). To facilitate the monitoring of wren nests, we placed 54 wooden nest boxes in the collection area with holes large enough to be accessed by cowbirds. These nest boxes were within or nearby mockingbird territories. Nest boxes are readily used by wrens and mostly preferred over natural cavities (Llambías and Fernández 2009). We monitored all nests constructed in nest boxes every 2 days during advanced construction and daily during laying. Although some wren breeding pairs nesting in natural cavities may have gone undetected, we are confident that we monitored the majority of the breeding pairs in the area as wrens prefer newly placed nest boxes over natural cavities in 95% of the cases (Llambías and Fernández 2009). Wrens accept all cowbird eggs (Mason 1986).

With the aim of studying the relationship between host availability and host use, for each nest, we recorded the starting date of the laying stage (appearance of the first host egg). To facilitate proper monitoring of the nest and determine the start of incubation stage, we also recorded the date of laying of the remaining host eggs, as well as the laying date of all parasite's eggs collected for molecular analyses (parasite eggs were marked with a permanent marker for later individual identification). In the cases where the nest was found with host eggs without incubation, we waited until the end of laying to calculate the starting date, assuming a clutch of four eggs for mockingbirds and five eggs for wrens. In this way, estimations of the starting date of each nest are not affected by host's egg losses. In the cases where the nest was found during the incubation stage (warm eggs), we determined the starting date from the hatching date of the first egg, by accounting for the average incubation period of each species (above). The exact locations of all nests were marked on a satellite image with Google Earth^®^ software (Google Inc. Mountain View, CA) (Fig. 2). For nests belonging to other hosts that we located occasionally, we simply recorded host species, geographical location and whether it was found during laying or incubation.

### Sample collection

Parasite's freshly laid eggs were artificially incubated (Yonar^®^, model 50/E) at 37.5 ± 1°C for 48 h to obtain adequate embryonic development and then frozen until processed (Strausberger and Ashley 2001), while eggs found with some degree of incubation (warm at touch, confirmed later with ovoscopy) were directly frozen (total eggs = 359). In cases where cowbird chicks were found in nests of the host species (*N* = 11), blood samples were taken via wing venipuncture and stored in lysis buffer (100 mmol/L Tris pH 8, 10 mmol/L NaCl, 100 mmol/L EDTA, 2% SDS). During the first two breeding seasons, we also captured 32 adults with walk‐in traps baited with millet (2008: six females and 10 males, 2009: seven females and nine males; sexed by plumage). The traps were placed in the same three plots used for egg collection. We took blood samples using the same procedure as in chicks and ringed individuals before release.

### Analysis of genetic data

For genetic analyses, embryonic tissue was extracted from the eggs and stored in DMSO buffer (20% v/v DMSO, 250 mmol/L EDTA, NaCl). DNA extraction of embryonic samples was performed following a standard protocol of dehydration and precipitation with ethanol and NaCl (Miller et al. 1988). Seven microsatellite loci designed for brown‐headed cowbirds were amplified using two multiplex‐touchdown polymerase chain reaction (PCRs): (1) CB1, CB12, and CB15 (Longmire et al. 2001), and (2) Ma*μ* 20 (Gibbs et al. 1997), Ma*μ* 25, Ma*μ* 29, and Dp*μ* 15b (Alderson et al. 1999). PCR amplifications for both sets of primers were performed in 10‐*μ*L reaction volumes using 20–60 ng of DNA template, 0.2 *μ*mol/L forward and reverse primers, 0.25 *μ*mol/L dNTPs, 2.5 mmol/L MgCl2, and 0.25 U Taq polymerase. Cycling temperatures were 95°C for 4 min then 10 cycles of 94°C for 30 sec, 55–53°C for 45 sec, and 72°C for 45 sec, then 35 cycles of 94°C for 30 sec, 53°C for 45 sec, and 72°C for 45 sec, finishing with 72°C for 40 min. The forward primer for each locus was fluorescently labeled and analyzed on an Applied Biosystems Model 3130xl Genetic Analyzer. Genotypes were assigned using Peak Scanner ^™^ v.1.0 (Applied Biosystems, Foster City, CA).

Genotypes of adult individuals were used to estimate population genetic parameters. The calculation of observed and expected heterozygosities and tests for departures from Hardy–Weinberg and linkage equilibrium were conducted with Genepop v. 4.0 (Raymond and Rousset 1995; Rousset 2008). Loci were checked for null alleles with Micro‐checker v. 2.2.3 (Van Oosterhout et al. 2004).

In order to analyze an individual female's laying behavior, we assigned eggs to particular females creating full‐sibling groups. To assess the statistical confidence for individual identification with our set of markers, we calculated the probability of identity PI_(ID)_ and the probability of identity between siblings PI_(SIB)_ (the probability that two individuals drawn at random from a population will have the same genotype at multiple loci, Waits et al. 2001) using the software Gimlet v. 1.2.3 (Valière 2002). Sibling group reconstruction was used to assemble sets of offspring that belonged to individual females which had also been fertilized by the same male, thus including only full‐siblings. Relatedness coefficients (*r*) among shiny cowbird samples were calculated using ML‐Relate (Kalinowski et al. 2006). Full‐siblings were identified for *r*‐values above the empirical cutoff value calculated with the software iRel v. 1.0 (Gonçalves da Silva and Russello 2011) following the procedure of Russello and Amato (2004). For egg pairs with full‐sibling *r*‐values, we tested specific hypotheses of full‐siblings versus half‐siblings and unrelated samples to discard full‐sibling relationships assigned by chance. Hypothesized relationships of full‐siblings were accepted for *P *> 0.99 (Marshall et al. 1998; Goodnight and Queller 1999; McPeek and Sun 2000; Kalinowski et al. 2006).

To confirm female identity, we also sequenced an 1120‐bp fragment of the mtDNA control region (Mahler et al. 2007) for individuals assigned to full‐sibling groups. Amplified products were sequenced on an Applied Biosystems Model 3130xl Genetic Analyzer and manually edited using CodonCode Aligner v. 5.0.1 (CodonCode Corporation, Centerville, MA). As full‐siblings must share the maternally inherited mitochondrial haplotype, we excluded those individuals that showed a different haplotype from the rest of the full‐sibling group. Full‐sibling groups composed of two members with different haplotypes were excluded from the analyses.

### Analysis of parasitism strategies

To analyze the spatial distribution of nests parasitized by the same female, we obtained coordinates of the nests from which her offspring was collected. Using the package “Geosphere” (Hijmans and Williams 2012) for R software (R Core Team, 2013), we calculated the distances between each pair of nests with one female's offspring. We then compared the distribution of distance data of parasitized nests with related eggs (for 17 sibling groups) and the distribution of distance data of parasitized nests with unrelated eggs using a nonparametric Kolmogorov–Smirnov test with InfoStat v. 2015 software (Di Rienzo et al. 2015). For the first group, 62 samples assigned to 17 females were included, while for the second group, 111 samples were included. This generated 100 distance values for samples of the first group and 5460 distance values for the second group.

For each cowbird female, we identified the host species utilized, and the breeding attempt at which they were parasitized. In cases of multiple and repeated parasitism, we determined the availability of nests appropriate for parasitism at that time, considering as “suitable” those nest at laying or early incubation stage (Fiorini et al. 2009). We also analyzed temporal laying patterns and calculated the maximum laying range for cowbird females in the study area (in number of days).

## Results

### Data collection and genetic data

In total, 316 nests were monitored during the three breeding seasons, including 220 mockingbird nests and 96 wren nests belonging to an average of 20 and 28 reproductive couples per season, respectively (Table 1). Mockingbird reproductive pairs had an average (±SD, standard deviation) of 2.8 (±0.3) breeding attempts per season versus 1.2 (±0.1) in wrens. Mean frequency of parasitism was 61.3% (±3.8) and 40.1% (±2.7) nests parasitized, and the intensity of parasitism was 2.2 and 1.2 eggs per nest, respectively (Table 1). Although climatic conditions differed among years, with very dry (2008) and very rainy (2009) seasons, parasitism frequencies and intensities per host remained fairly constant. We collected 402 individual cowbird samples during the three breeding seasons, corresponding to 311 offspring samples taken from mockingbird nests, 53 from wren nests, 6 from rufous‐collared sparrows *Z. capensis* nests, and 32 adult cowbird samples.

**Table 1:** Details of nests monitored in the three breeding seasons (2008–2010) by host species

	2008					2009					2010				
	Bp	*N*	*N*_P_	*I*	*R*	Bp	*N*	*N*_P_	*I*	*R*	Bp	*N*	*N*_P_	*I*	*R*
Chalk‐browed mockingbird	24	77	50	2.3 ± 1.8	1–11	22	68	42	2.5 ± 1.6	1–6	22	75	43	1.8 ± 1.0	1–5
House wren	25	35	14	1.2 ± 0.4	1–2	41	48	15	1.2 ± 0.4	1–2	20	21	9	1.2 ± 0.4	1–2
Rufous‐collared sparrow	2	2	2	1.5 ± 0.0	1–2	2	2	2	1.5 ± 0.0	1–2					
Number of collected samples	134					148					88				

Bp: number of breeding pairs; *N*: number of nests found; *N*_P_: number of parasitized nests; *I*: intensity of parasitism (mean number ± standard error of parasite eggs per parasitized nests) ; *R*: minimum and maximum number of cowbird eggs found in a nest.

We were able to genotype 198 offspring samples and the 32 adult samples. Unfortunately, for the remaining eggs, we either could not ensure embryonic development despite pre‐incubation for 48 h, or the extracted DNA was not sufficient for successful amplification or genotyping. Genetic variability data for the adults' samples are shown in Table 2. Locus Dp*μ* 15b presented many amplification problems and was subsequently eliminated for genotyping. The locus CB1 showed deviation from Hardy–Weinberg equilibrium and evidence of null alleles and was thus excluded from kinship analyses. Probabilities of identity for the remaining five loci were PI_(ID)_ = 3.2E^−08^ and PI_(sib)_ = 4.2E^−03^, below the thresholds suggested by Waits et al. (2001; PI_(ID)_ < 0.001 and PI_(sib)_ < 0.05).

**Table 2:** Characteristics of the six microsatellite loci genotyped from a sample of 32 adult shiny cowbirds

	*K*	Range (pb)	*H*_o_	*H*_e_	*P*‐value_EHW_
CB. 1	12	203–267	0.688	0.884	**0.0003**
CB. 12	14	152–248	0.955	0.905	0.7709
CB. 15	8	241–273	0.938	0.844	0.9831
Ma*μ* 20	14	120–172	0.903	0.840	0.2274
Ma*μ* 25	10	122–154	0.917	0.850	0.2567
Ma*μ* 29	11	115–179	0.708	0.811	0.1053

*K*: number of alleles and its molecular weight range in base pairs (pb); *H*_o_: observed heterozygosity; *H*_e_: estimated heterozygosity; *P*‐value_EHW_: departure from Hardy–Weinberg equilibrium. Bold value indicates statistical significance.

### Kinship analysis

Allele frequencies used for kinship analysis were calculated from the adult samples. To determine the empirical cutoff value with software iRel, *r*_QG_ showed greater discriminatory power between adjacent categories of kinship and the allocation of full‐siblings was above *r* = 0.363. Using this cutoff value, pairs of full‐siblings were identified and subject to hypothesis testing. There were 87 eggs assigned to 26 groups of full‐siblings, with an average of 3.3 eggs per group (minimum 2–maximum 7). After mtDNA analysis of these individuals, we found 8 of the haplotypes found by Mahler et al. (2007). Then, 25 samples had to be excluded, leaving 17 groups of full‐siblings (2008: *N* = 10, 2009: *N* = 5, 2010: *N *=* *2). No full‐sibling groups containing members from different years were established, so each group was considered to be independent (i.e., from a different female). Remaining offspring (*N *=* *136) could not be assigned to full‐sibling groups and thus were not further considered for analyses on laying strategies.

### Spatial and temporal nest use

**Figure 3:**
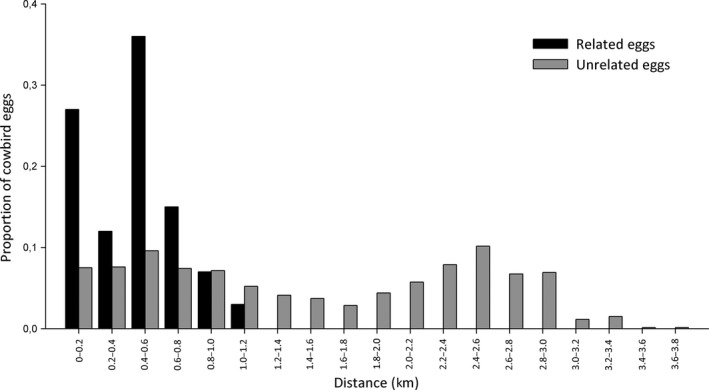
Spatial distribution of nests parasitized with eggs of the same female (black) and with unrelated eggs (gray). Kolmogorov–Smirnov, KS = 0.6, *P* < 0.01.

**Figure 4:**
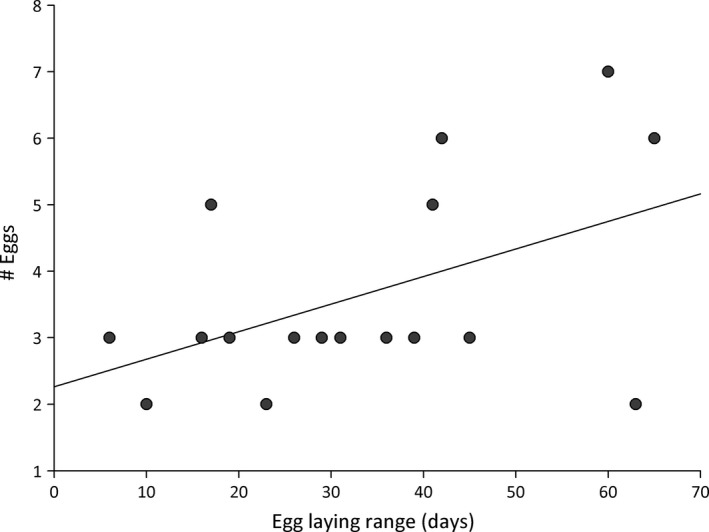
The number of eggs in relation to seasonal laying range (range of days between the date of the first and the last egg assigned to each female) for the 17 females studied (Simple linear regression: *R* ^2^: 0.23, *N* = 17, *P* = 0.48, *P* = 0.049).

The spatial distribution analysis showed that eggs belonging to full‐sibling groups were found in nests that were closer than nests with unrelated eggs (Kolmogorov–Smirnov, KS = 0.6, *P* < 0.01, Fig. 3). Nests with related eggs were found at a median distance of 440 m mostly within the same collection area (Fig. 2). In contrast, eggs laid by different females showed a bimodal distribution, corresponding to eggs laid within the same area and in different collection areas (Fig. 2). In our study area, the laying season lasted 86 ± 9.5 days, beginning the first days of October and finishing by mid‐January. Laying periods among identified females mostly overlapped during the breeding season. We found a maximum individual laying period of 65 days (Fig. 4) and a maximum laying rate of 0.5 eggs per day, corresponding to three eggs found for a female in a time range of 6 days (Fig. 4). This value is somewhat lower than the one documented by Kattan (1993) of 0.66 eggs per day.

### Host selection

**Figure 5:**
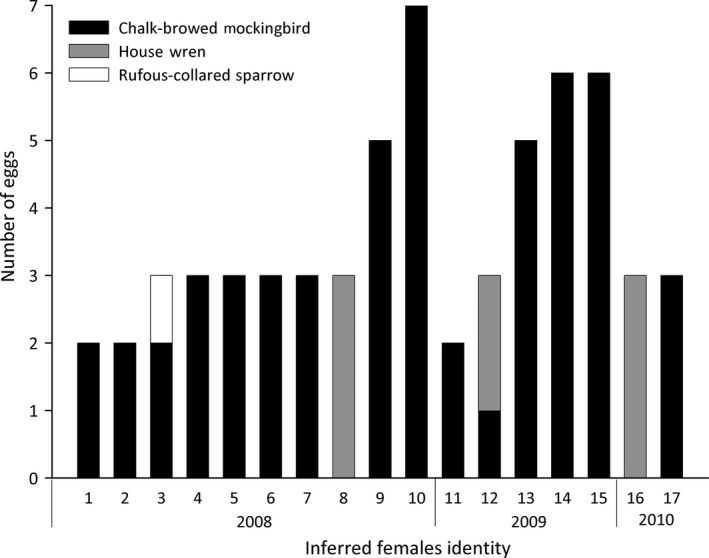
Detail of the hosts used by each of the 17 females studied: chalk‐browed mockingbird (black), house wren (gray), and rufous‐collared sparrow (white).

Of the 17 different full‐sibling groups, 15 were composed of eggs found in nests of a single host species (13 in mockingbird nests and two in wren nests, Fig. 5). The remaining two full‐sibling groups were composed of eggs from nests of more than one host species: one with eggs from mockingbird and wren nests and the other one from mockingbird and rufous‐collared sparrow nests (Fig. 5).

Considering that full‐sibling groups represent different females, we studied individual parasite females laying behavior analyzing host selection and nest use. When analyzing nest availability for the two females that used nests of more than one host species (female 3 and 12, Fig. 5), we found that nests of both hosts were available during laying and early incubation for each parasitism event, thus indicating that use of another host was not related to an absence of nests of the other one.

**Figure 6:**
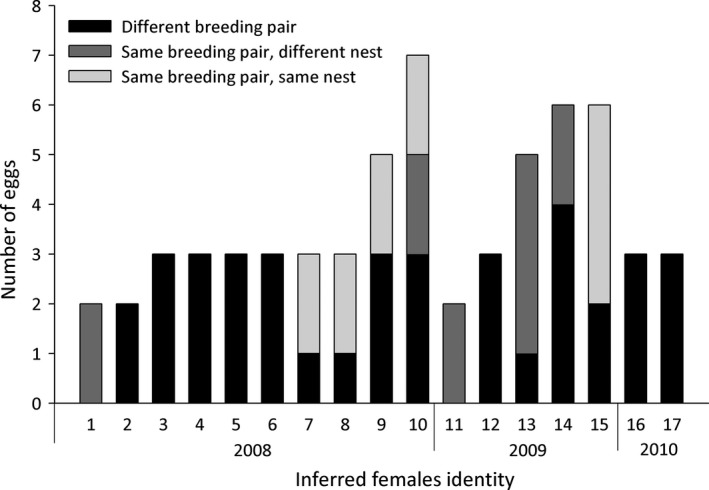
Detail of the nests used by each of the 17 females studied: different breeding pair (black), same breeding pair – different nest (dark gray) and same breeding pair – same nest (light gray).

For eight females, eggs were found in nests of different breeding pairs (Fig. 6). For nine females, some eggs were found in nests of the same breeding pair. Four cowbird females parasitized only different breeding attempts, which were successive in some cases and interspersed in others, while for five females eggs were found in the same nest (Fig. 6). It is noteworthy that one female laid twice in the same nest of two different breeding pairs. This indicates that at least five of 17 females engaged in repeated parasitism. Use of the same nest was made with an interval of 1–4 days. A detailed evaluation of nest availability at the date when the female used the same nest for the second time showed that there were other options for laying: At least seven available nests were in the proximity of the nest used by female 7, four nests for females 8 and 10, and five nests for female 9. In the case of female 15, that showed repeated parasitism twice, there were three and one other suitable host nests available, respectively, at the time of laying repeatedly.

## Discussion

Our results show that the 17 studied laying Shiny Cowbird females were mostly faithful to a particular laying area and showed a preference for one of the monitored host species. Also, they did not avoid using nests previously parasitized by themselves or by another female. Nests parasitized by the same female were within a limited area, showing individual laying site fidelity. Eggs laid by the same female were found <1 km apart, with a mean distance of 488 m. These results are consistent with the findings of Scardamaglia and Reboreda (2014) who studied the movements of parasitic females during the reproductive season using radio telemetry and found that the area used by a female was on average 25 ha. Taken together, these findings show that cowbird females use relatively constant areas for nest searching and parasitism and that they maintain this area throughout the breeding season.

Use of the same breeding area by several females makes multiple parasitism very common at this study site (Gloag et al. 2012; also see Stevens et al. 2013). We found a high proportion of parasitized nests with more than one parasitic egg (57% in mockingbirds and 21% in wrens). The use of the same area during the entire reproductive season led five parasitic females to parasitize the same breeding pair several times at different reproductive attempts (Fig. 6). Moreover, we found five females that engaged in repeated parasitism. This laying strategy has not been previously reported for this parasite species (Kattan 1997; Lyon 1997; Gloag et al. 2014). Multiple parasitism imposes a cost to parasitic females, because it increases predation risk, egg puncture by other cowbird females and the competition among gregarious nestlings. Costs from this last reason will be even higher when multiple parasitism involves the offspring of the same female. However, this strategy might be beneficial when the costs of multiple parasitism are lower than those used to locate and monitor additional nests (Gloag et al. 2014) or exceeded by the benefits of choosing a host that can successfully raise multiple parasitic chicks (Martínez et al. 1998), especially when host availability is low (Lyon 1997). On the one hand, both host species have been shown to raise more than one parasite chick successfully (Fraga 1985; Fiorini 2007; Tuero et al. 2007), although mockingbirds have higher success than wrens in doing so (Fraga 1985; Fiorini 2007) as mortality rates are higher in the latter when more than one parasite is in the nest (Kattan 1997; Tuero et al. 2007). On the other hand, the presence of additional nest mates might enhance feeding rates of the foster parents delivered to their own genetic chicks, too (Kilner et al. 2004; Gloag et al. 2012). We found that multiple parasitism was higher in mockingbirds than in wrens (57% vs. 21%), although it is not clear from our data whether this is adaptive host use or a consequence of greater nest detectability in the mockingbirds (Strausberger 1998; Strausberger and Ashley 2003; Rivers et al. 2012).

In the case of repeated parasitism, we found that when the female used the same nest that she parasitized before, there were three to five other nests of the host available at the time in five of the six cases. Just for one event of repeated parasitism only one other nest was available. Although we cannot discount that some available nests were not found by parasitic females, evidence suggests that females do not actively avoid parasitizing the same nest. One strategy could be random use among available nests within the laying area. If this was the case, it might be possible that repeated parasitism is a consequence of lower nest density, where lower nest densities increase the probability of using the same nest. Accordingly, in comparisons with the study site of Gloag et al. (2014), who did not find evidence of repeated parasitism, our site had about half the nest density (approx. 0.18 nest/ha vs. 0.08 nest/ha, respectively).

The maximal daily laying rate found at our study site was 0.5 eggs/day, somewhat less than the laying rate found for a population of this species in Central America, which was estimated in 0.6 eggs per day (Kattan 1993; Rueda‐Cediel et al. 2008), but quite similar to populations of brown‐headed cowbirds of Texas and Florida (0.57 and 0.56 eggs per day, respectively; Reetz 2008). Considering geographical differences in breeding season duration, we expected higher laying rates at our more temperate study site (Reetz 2008). Similar values might be related to physiological constraints or to a dependency on nutritional factors that might account for regional differences in reproductive values (Payne 1965; Scott and Ankney 1980; Fleischer 1985).

We also found that females parasitize the hosts within their laying area along the entire breeding season, but we were unable to determine whether laying is constant throughout the season or whether females lay their eggs in bouts with resting periods in‐between, as well as whether the laying pattern is generalized or varies among females. Although we found that three females laid throughout the season with longer intervals (approx. every 10–13 days) and other females laid every other day, we cannot discard that these differences are related to incomplete sampling.

Along three breeding seasons, we observed that parasitism frequencies and intensities were higher in mockingbird than in wren nests. These values did not change even in the 2009 season, when we found many more house wren nests and reproductive couples (Table 1). This suggests a preference of shiny cowbird females for mockingbird nests, supporting the hypothesis that females prefer certain hosts at population level (Mason 1986; De Mársico et al. 2010) and only parasitize a small fraction of the available hosts in a community, not parasitizing or only infrequently parasitizing a large proportion of available hosts (De Mársico et al. 2010). The majority of the females only used nests of mockingbirds for laying while one only parasitized house wrens, indicating a preference for one host species. Just two females were found to use nests of more than one host. Parasitism of more than one host was also reported by Gloag et al. (2014). Additionally, mtDNA distribution patterns suggested the existence of host preference in the shiny cowbird but also frequent host switches (Mahler et al. 2007; Domínguez et al. 2015). Taken together, these results suggest that the preference for one host might be partial or flexible according to the conditions. Although the use of alternative hosts might be underestimated because some hosts were not sampled in our study area and some eggs might have been laid outside the sampling area or even rejected by mockingbirds (and hence not collected), we are confident that we collected a representative sample of the eggs as females' laying areas are limited and the two hosts that were sampled are among the most abundant (Mason 1985) and have the highest parasitism frequencies. Other abundant host species have considerably lower parasitism frequencies by shiny cowbirds (Mason 1985; Mason 1986) or are even not available at this study plot, like the yellow‐winged blackbird *Agelasticus thilius* or the brown‐and‐yellow marshbird *Pseudoleistes virescens*, which nest in wetlands.

As found for the brown‐headed cowbird (Alderson et al. 1999; Woolfenden et al. 2003; Strausberger and Ashley 2005), shiny cowbird females of our population showed both host‐specialist and host‐generalist laying strategies. Although we cannot be sure that we sampled all eggs from each female, we observed that some of them had a preference for one host species (females 9, 10, 13, 14, and 15, Fig. 5). This host‐specialist behavior is associated with a preference for a host which is not expected to be absolute or exclusive (Tversky 1969). For the brown‐headed cowbird *M. ater*, several studies showed flexible laying behavior that was related to parasite female density (Alderson et al.^1999^; Hahn et al.^1999^; McLaren et al.^2003^; Ellison et al. 2006; Rivers et al. 2012) or host nest availability (Strausberger and Ashley 2003; Rivers et al. 2012). Also, the Horselfield's bronze cuckoo *Chalcites basalis* shows plasticity in host and habitat preference, allowing the use of secondary hosts when the preferred one is spatially or temporally absent (Langmore and Kilner 2007). In our population, host nest availability does not account for laying decisions: For both females that used more than one host, we assessed the availability of nests of the other species at the day of laying and found that nests of both host species were available at similar proportions in all cases.

One explanation for the use of nests of alternative hosts might be social learning, with females following other females to detect available and suitable nests for parasitism. Radio‐tracking studies in our study area (Scardamaglia and Reboreda 2014) showed that females roost communally and leave the roosts in groups before dawn. Various females (up to 4) approach host nests simultaneously for laying (Fraga 1985; Gloag et al. 2014) which suggests that females might detect an available nest by following other females within their laying territory. Although the mechanism underlying specialist or generalist individual host use is not clear, our results are consistent with the absence of complete maternal lineage sorting of brood parasitism between different host species, as reported by Mahler et al. (2007). These results indicate that coevolutionary interactions between shiny cowbirds and their hosts are not restricted to one host‐specific group of cowbirds and its particular host but that interactions with hosts rather involve the cowbird population as a whole. This makes monitoring of endangered species used as hosts particularly important (López‐Ortiz et al. 2006), as parasitism pressure alone or combined with other causes could drive small populations to local extinctions (Domínguez et al. 2014). Future work could manipulate host nest availability experimentally to assess the role of individual preferences versus ecological constraints in host species selection and nest use by individual brood parasitic females in this and other species.

## Ethical standards

This research was conducted in accordance with relevant Argentinean regulations (Law of Conservation of Wild Fauna), under the permit issued to JCR, University of Buenos Aires.

## Conflict of Interest

None declared.
